# The influence of progesterone on bovine uterine fluid energy, nucleotide, vitamin, cofactor, peptide, and xenobiotic composition during the conceptus elongation-initiation window

**DOI:** 10.1038/s41598-019-44040-6

**Published:** 2019-05-22

**Authors:** Constantine A. Simintiras, José M. Sánchez, Michael McDonald, Patrick Lonergan

**Affiliations:** School of Agriculture and Food Science, University College Dublin, Belfield, Dublin 4 Ireland

**Keywords:** Reproductive biology, Developmental biology, Metabolomics

## Abstract

Conceptus elongation coincides with one of the periods of greatest pregnancy loss in cattle and is characterized by rapid trophectoderm expansion, commencing ~ Day 13 of pregnancy, *i.e*. before maternal pregnancy recognition. The process has yet to be recapitulated *in vitro* and does not occur in the absence of uterine gland secretions *in vivo*. Moreover, conceptus elongation rates are positively correlated to systemic progesterone in maternal circulation. It is, therefore, a maternally-driven and progesterone-correlated developmental phenomenon. This study aimed to comprehensively characterize the biochemical composition of the uterine luminal fluid on Days 12–14 – the elongation-initiation window – in heifers with normal *vs*. high progesterone, to identify molecules potentially involved in conceptus elongation initiation. Specifically, nucleotide, vitamin, cofactor, xenobiotic, peptide, and energy metabolite profiles of uterine luminal fluid were examined. A total of 59 metabolites were identified, of which 6 and 3 displayed a respective progesterone and day effect, whereas 16 exhibited a day by progesterone interaction, of which 8 were nucleotide metabolites. Corresponding pathway enrichment analysis revealed that pyridoxal, ascorbate, tricarboxylic acid, purine, and pyrimidine metabolism are of likely importance to to conceptus elongation initiation. Moreover, progesterone reduced total metabolite abundance on Day 12 and may alter the uterine microbiome.

## Introduction

One-third of viable bovine blastocysts are estimated to fail to elongate^1–3^, rendering them unable to secrete sufficient interferon tau (IFNτ), the pregnancy recognition signal^4–7^, resulting in pregnancy failure on account of a lack of uterine oxytocin receptor upregulation for luteolysis prevention^6,8^. Any insights into the mechanisms driving conceptus elongation, therefore, offer scope to improve ruminant fertility.

Conceptus elongation is a maternally-driven and progesterone-correlated developmental phenomenon^9^; it can neither be recapitulated *in vitro*^10^ nor can occur *in vivo* in uterine gland absence^11^. Moreover, the rate of conceptus elongation is positively related to maternally circulating progesterone (P4)^12,13^, though this relationship is indirect – *in vitro* derived embryos transferred on Day 7 into heifers previously (Days 3–6) supplemented with P4 exhibit advanced elongation on Day 14, regardless of identical systemic P4 during *in utero* embryo development^14^.

Whilst the amino acid^15–20^, carbohydrate^20–23^ and lipid^2,24,25^ composition of bovine uterine luminal fluid (ULF) has been interrogated to some degree, analyses extending to additional biochemicals (*e.g*. nucleotide, cofactor and vitamin, xenobiotic, peptide, and energy metabolites) have received very little attention, if any, despite their known importance to, and/or potential impact on, central cellular processes.

Nucleotides serve a variety of functions such as in substrate activation and anabolism, in addition to being necessary for DNA replication and RNA production to support protein synthesis^26^. Cofactors and vitamins assist a plethora of enzymatic reactions by facilitating electron and acyl group transfer, amongst others^27,28^; energy metabolites are typically involved in terminal substrate oxidation, such as in the tricarboxylic acid (Krebs) cycle^29^, and peptides have been shown to act as hormones and signalling molecules^30^ in addition to antibiotics, such as microcins^31,32^. Lastly, xenobiotics are foreign substances (*e.g*. drugs, pollutants, food additives, hydrocarbons, and pesticides) which may perturb physiological function^33^.

We recently showed that the amino acid, carbohydrate^20^, and lipid^25^ composition of bovine ULF on Days 12–14 post-estrus was affected by day and P4 supplementation, revealing several metabolites of likely importance to the process of conceptus elongation initiation. Given the biological significance of the aforementioned metabolites (nucleotides, vitamins, *etc*.), coupled with the lack of information surrounding their presence in ULF, our hypothesis was that a high-throughput metabolomic profiling of these ULF samples would identify several additional molecules which likely influence conceptus elongation. The specific aim, therefore, was to analyse the ULF obtained on Days 12–14 from cycling heifers with normal *vs*. high P4 in circulation, a model known to advance the rate of conceptus elongation^12,13,34^.

## Results

P4-releasing intravaginal device (PRID) insertion on Day 3 elevated (*P* ≤ 0.05) serum P4 on Day 5 (3.17 ± 0.341 *vs*. 1.53 ± 0.163 ng/ml), as determined by a two-way ANOVA coupled with a Holm-Sidak non-parametric *post hoc*. This difference was not apparent by Days 12–14. Moreover, no difference in systemic P4 was observed between Days 12–14 within the normal and high P4 groups, as described in Simintiras *et al*.^20^.

**Figure 1:**
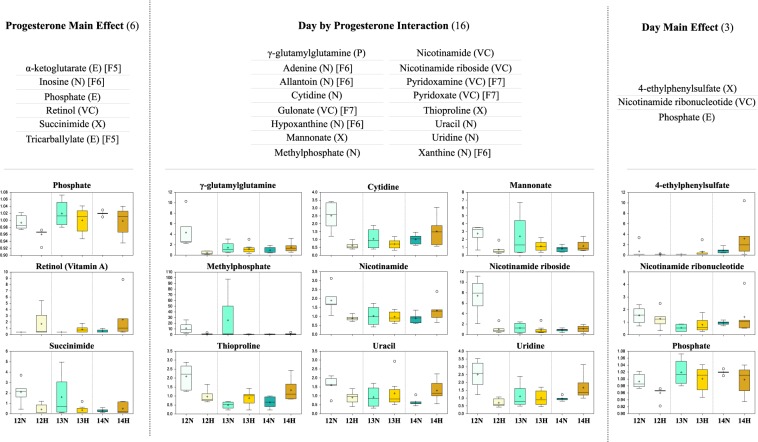
The identified metabolites, and their respective pathways, showing a progesterone (P4) main effect (left), a day main effect (right), and/or a day by P4 interaction (centre). Phosphate showed both a progesterone and day main effect but not a day by P4 interaction. Day and/or P4 main effects are discounted where a day by P4 interaction is displayed. Corresponding metabolite scaled intensities are also provided, wherein the central horizontal line represents the median value with outer boundaries depicting upper and lower quartile limits. The y-axes are the relative metabolite concentrations. Error bars depict the minimum and maximum distributions, with a cross (+) representing the mean value and a white circle (○) the extreme data point. Square brackets denote the figure in which the scaled intensities for the metabolite is provided. Abbreviations: Fig. 5 [F5], Fig. 6 [F6], Fig. 7 [F7], energy metabolite (E), nucleotide metabolite (N), vitamin and cofactor metabolite (VC), xenobiotic (X), peptide (P), Day 12 Normal P4 (12N), Day 12 High P4 (12H), Day 13 Normal P4 (13N), Day 13 High P4 (13H), Day 14 Normal P4 (14N), and Day 14 High P4 (14H).

A total of 59 metabolites were identified in this study, spanning 19 pathways; 11 metabolites (18.6%) related to energy metabolism, 19 (32.2%) to nucleotide, 12 (20.3%) to cofactor and vitamin, 15 (25.4%) to xenobiotic, and 2 metabolites (3.4%) related to peptide metabolism (Table 1). Five metabolites (8.5%) displayed a P4 main effect, *i*.*e*. differed in abundance between high *vs*. normal P4 heifers, irrespective of day, whilst 2 (3.4%) exhibited a day main effect, *i*.*e*. were temporally dynamic independently of P4, and 1 metabolite (1.7%) – phosphate – was affected by both day and P4. Sixteen metabolites (27.1%) displayed a day by P4 interaction, of which half are implicated in nucleotide metabolism (Fig. 1).

**Table 1:**
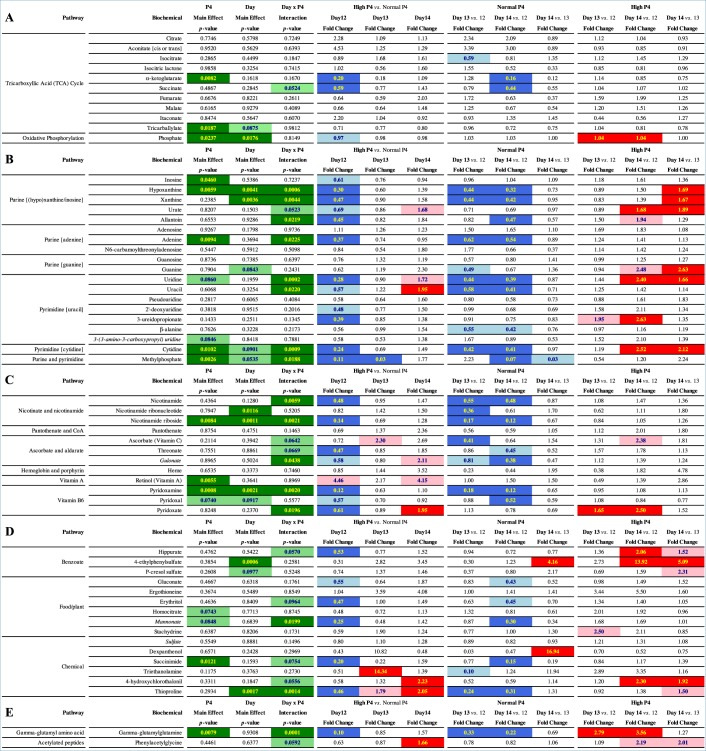
Detected metabolites involved in (*A*) energy, (*B*) nucleotide, (*C*) vitamin and cofactor, (*D*) xenobiotic, and (*E*) peptide metabolism.

Regarding day and/or progesterone main effects and/or day by progesterone interactions (first three columns), light green shading highlights a trend towards a significant (0.05 < p < 0.10) effect, whereas dark green shading highlights a statistically significant (p ≤ 0.05) effect with individual p-values provided within cells. Remaining columns comprise individual metabolite fold changes: dark blue shading indicates a significant (p ≤ 0.05) decrease (metabolite ratio < 1.0) between groups shown, whereas light blue depicts a decreasing trend (0.05 < p < 0.10). Conversely, dark red shading indicates a significant (p ≤ 0.05) increase (metabolite ratio ≥ 1.0) between groups shown with light red depicting an increasing trend (0.05 < p < 0.10). Non-coloured cells and text indicate the mean fold-change value was not significantly different for that comparison. Italics denotes predicted metabolites. Abbreviations: progesterone (P4) and coenzyme A (CoA).

**Figure 2:**
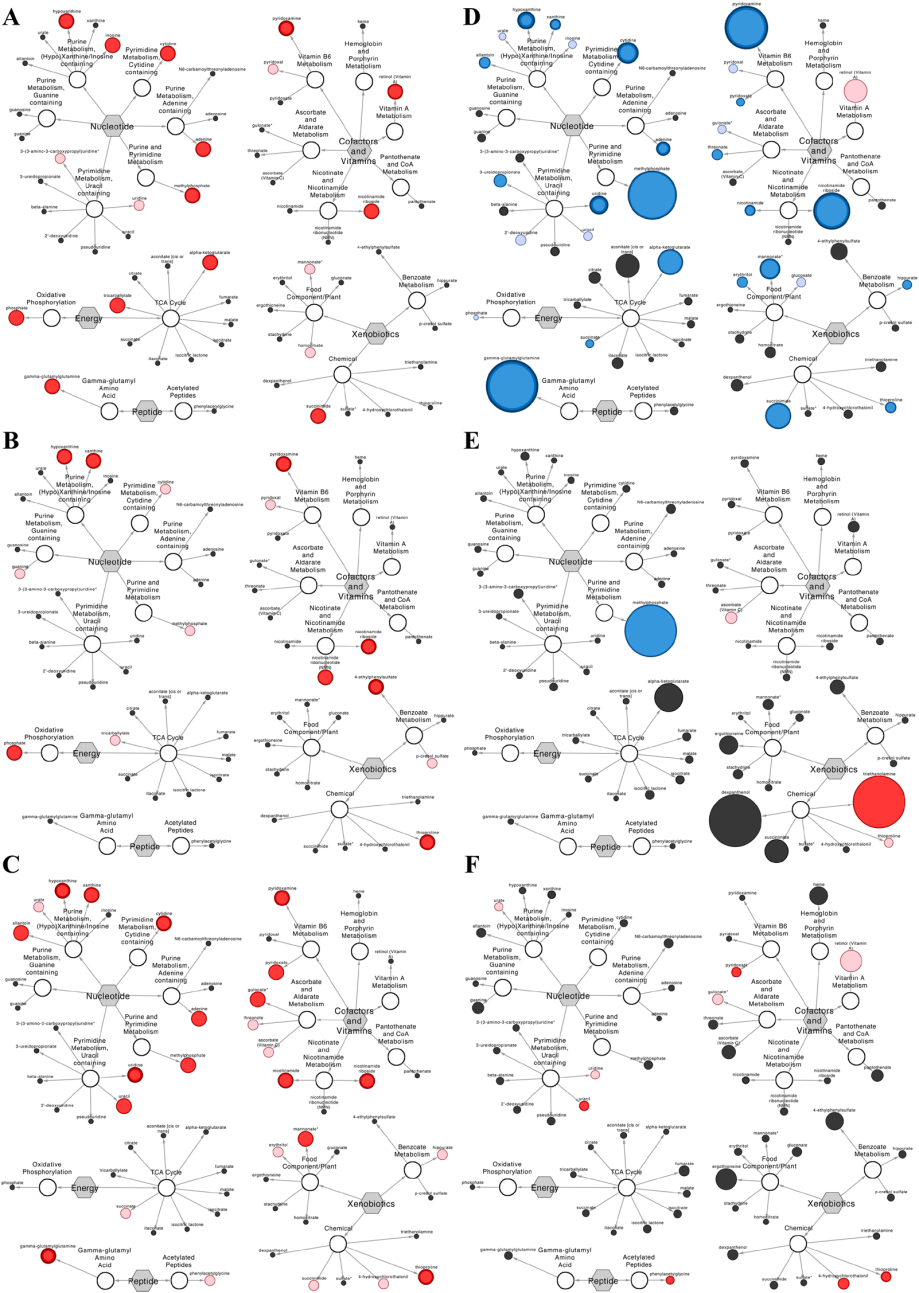
Network comparison of metabolite relative flux in uterine luminal fluid. Networks on the left-hand side show biochemicals which displayed (**A**) a progesterone (P4) main effect, (**B**) a day main effect, and/or (**C**) a day by P4 interaction – *i.e*. concentrations differed between groups (normal *vs*. high P4) at different times (Days 12 *vs*. 13 *vs*. 14). Significance is represented by node colour and diameter combined: a large dark red node indicates an (p ≤ 0.05) effect/interaction (node border thickness is inversely proportional to the magnitude of the p-value), whereas a medium light red node depicts a trend (0.05 < p < 0.10) towards an effect/interaction, and a small black nodes depict a lack of significance. Networks on the right-hand side compare metabolite flux magnitude in the uterine luminal fluid of normal *vs*. high P4 heifers on Days (**D**) 12, (**E**) 13, and (**F**) 14. Here, node diameter is proportional to the fold change observed, and node colour represents the significance of the change: dark red depicting a significant (p ≤ 0.05) increase, light red highlighting an increasing trend (0.05 < p < 0.10), dark blue denoting a significant (p ≤ 0.05) decrease, and light blue depicting a decreasing trend (0.05 < p < 0.10). Black nodes depict a lack of a statistically significant flux. In addition to node colour, node border thickness is inversely proportional to the magnitude of the p-value.

Regarding the corresponding pathways of the identified molecules, the most enriched by P4 (*n* = 13, *N* = 59) – whereby E is the pathway enrichment, *k* refers to the number of significantly altered metabolites per pathway, *m* denotes the total number of detected molecules per pathway, *n* depicts the number of significantly altered metabolites in the comparison, and *N* represents the total identified metabolites in the study – were gamma-glutamyl amino acid (E = 4.5; *k* = 1, *m* = 1), oxidative phosphorylation (E = 4.5; *k* = 1, *m* = 1), pyrimidine [cytidine] (E = 4.5; *k* = 1, *m* = 1), purine and pyrimidine (E = 4.5; *k* = 1, *m* = 1), vitamin A (E = 4.5; *k* = 1, *m* = 1), and purine [(hypo)xanthine/inosine] (E = 1.8; k = 2, m = 5) metabolism (Fig. 2A). Pathways most enriched by day (*n* = 8, *N* = 59) were oxidative phosphorylation (E = 7.4; *k* = 1, *m* = 1), nicotinate and nicotinamide (E = 4.9; *k* = 2, *m* = 3), purine [(hypo)xanthine/inosine] (E = 3.0; *k* = 2, *m* = 5), vitamin B6 (E = 2.5; *k* = 1, *m* = 3), benzoate (E = 2.5; *k* = 1, *m* = 3), and chemical (E = 1.2; *k* = 1, *m* = 6) metabolism (Fig. 2B). However, the most enriched metabolic pathways in terms of day by P4 interaction (*n* = 16, *N* = 59) were gamma-glutamyl amino acid (E = 3.7; *k* = 1, *m* = 1), pyrimidine [cytidine] (E = 3.7; *k* = 1, *m* = 1), purine and pyrimidine (E = 3.7; *k* = 1, *m* = 1), nicotinate and nicotinamide (E = 2.5; *k* = 2, *m* = 3), vitamin B6 (E = 2.5; *k* = 2, *m* = 3), purine [(hypo)xanthine/inosine] (E = 2.2; *k* = 3, *m* = 5), purine [adenine] (E = 1.2; *k* = 1, *m* = 3), ascorbate and aldarate (E = 1.2; *k* = 1, *m* = 3), and pyrimidine [uracil] (E = 1.1; *k* = 2, *m* = 7) metabolism (Fig. 2C).

**Figure 3:**
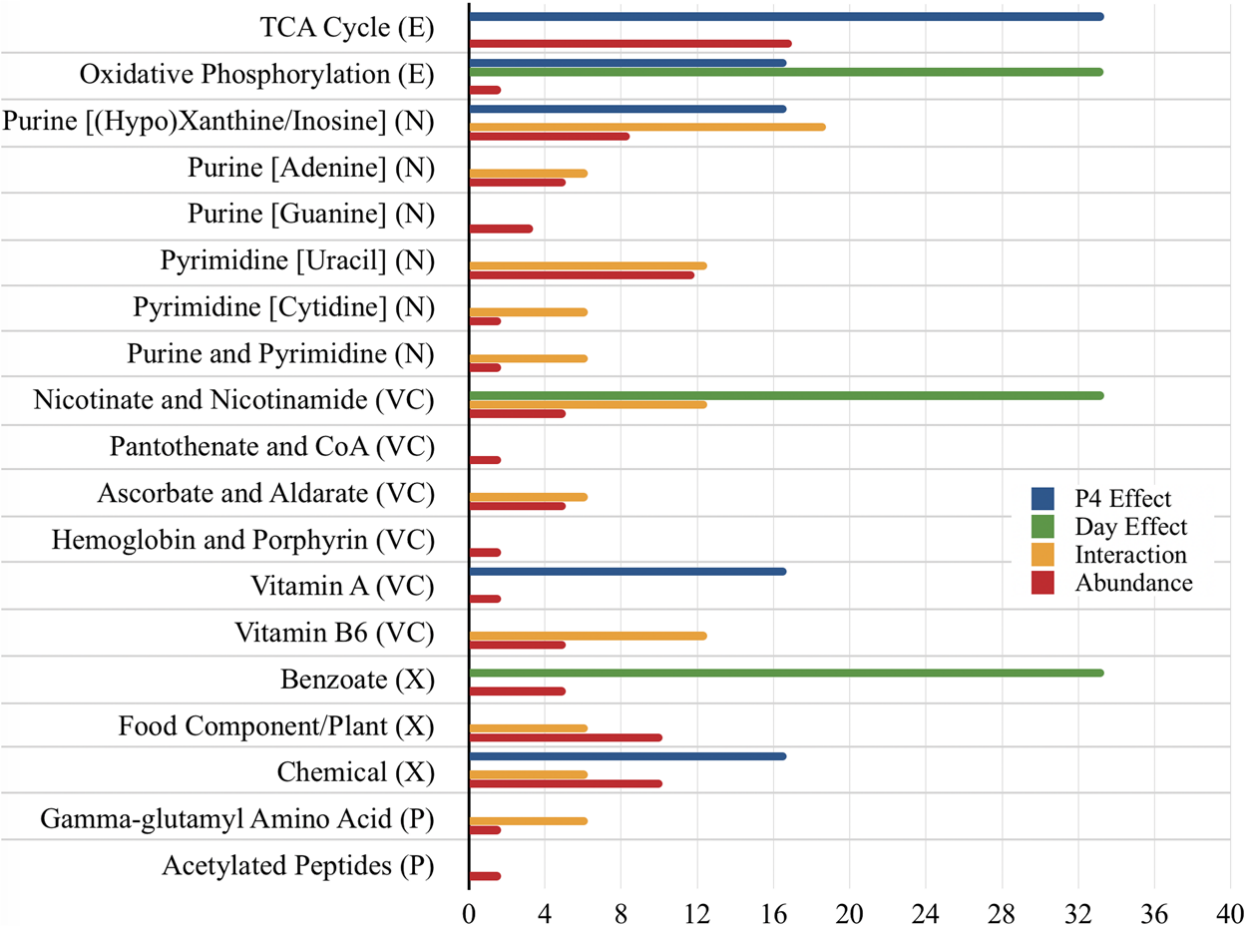
The contribution (%) of each sub-pathway to total (*a*) progesterone (P4) effects – *i.e*. the proportion of corresponding metabolites which were hormonally responsive, (*b*) day effects – *i.e*. the proportion of corresponding metabolites which were temporally dynamic, (*c*) day by P4 interactions – *i.e*. the proportion of corresponding metabolites whose concentrations differed between groups (normal *vs*. high P4) at different times (Days 12 *vs*. 13 *vs*. 14), and (*d*) abundance – *i.e*. the size of the sub-pathway. Abbreviations: progesterone (P4), super-pathways: energy metabolite (E), nucleotide metabolite (N), vitamin and cofactor metabolite (VC), xenobiotic (X), and peptide (P).

Considering main effect or interaction proportions, which are less susceptible to ‘single-metabolite’ pathway bias than enrichment analyses, pathways representing most P4 effects were: tricarboxylic acid cycle (33.3%), oxidative phosphorylation (16.7%), purine [(hypo)xanthine/inosine] (16.7%), vitamin A (16.7%), and chemical (16.7%) metabolism. Similarly, pathways exhibiting most day effects were: oxidative phosphorylation (33.3%), nicotinate and nicotinamide (33.3%), and benzoate (33.3%) metabolism; whereas pathways displaying the greatest proportion of day by P4 interactions were: purine [(hypo)xanthine/inosine] (18.8%), pyrimidine [uracil] (12.5%), nicotinate and nicotinamide (12.5%), and vitamin B6 (12.5%) (Fig. 3). Also provided in Fig. 3 is the relative abundance of each pathway (*i.e*. the number of metabolites constituting the pathway as a proportion of all identified metabolites).

P4 supplementation, moreover, reduced (p < 0.05) the concentrations of 21 metabolites on Day 12 (Fig. 2D). In contrast, the concentrations of just 2 metabolites differed on Day 13 in high *vs*. normal P4 heifers (Fig. 2E), including the greatest individual metabolite flux observed – a 14.34-fold increase in triethanolamine (Table 1D). On Day 14, however, P4 supplementation elevated the uterine luminal abundance of 5 metabolites (Fig. 2F); these were 4-hydroxychlorothalonil, phenylacetylglycine, pyridoxate, thioproline, and uracil.

**Figure 4:**
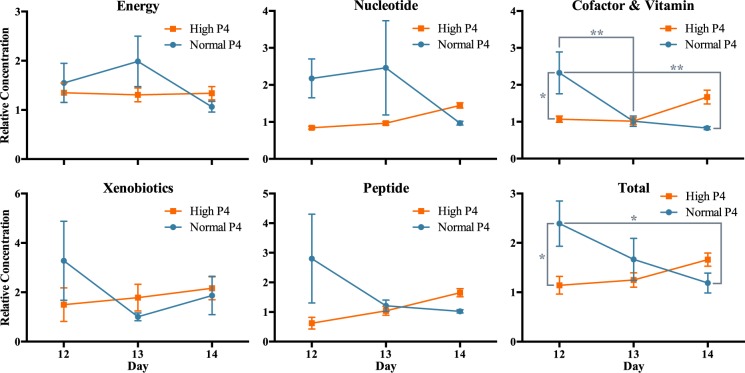
Relative concentrations (±SEM) of energy (n = 11), nucleotide (n = 19), cofactor and vitamin (n = 12), xenobiotic (n = 15), and peptide (n = 2) metabolites in the uterine luminal fluid of high and normal progesterone (P4) animals on Days 12, 13, and 14, wherein ** represents p ≤ 0.001 and * represents p ≤ 0.05 – as determined by two-way analysis of variance coupled with a Holm-Sidak non-parametric *post hoc* test.

Metabolite analysis by super-pathway revealed that cofactor and vitamin metabolite abundance in the uteri of normal P4 heifers exhibited the greatest variability, decreasing on Days 13 and 14 relative to 12. Moreover, when considering all metabolites combined, P4 supplementation supressed their abundance in ULF on Day 12 (Fig. 4).

**Figure 5:**
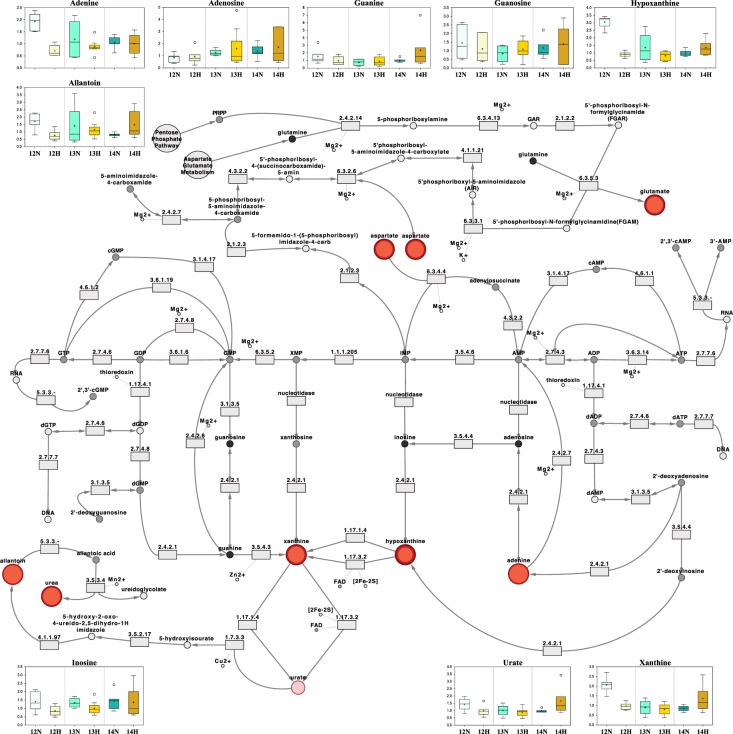
The purine metabolic pathway surrounded by the scaled intensities of relevant biochemicals. Biochemical node colour corresponds to day by progesterone (P4) interaction – dark red indicates a significant (p ≤ 0.05) interaction (node border thickness is inversely proportional to the magnitude of the p-value). Within these statistically significant nodes, node diameter is correlated to metabolic hierarchy (cofactor and intermediate metabolite nodes are smallest, followed by by-products, and central metabolites). Light red depicts an increasing trend (0.05 < p < 0.10), black depicts an identified amino acid which did not exhibit a day by progesterone interaction, and grey represents a biochemical present in the metabolic library but not detected in this study. Box plots: The y-axes are the relative metabolite concentrations, with the central horizontal line representing the median value with outer boundaries depicting the upper and lower quartile limits. Error bars depict the minimum and maximum distributions, with + representing the mean value and ○ the extreme data point. Abbreviations: Day 12 Normal P4 (12N), Day 12 High P4 (12H), Day 13 Normal P4 (13N), Day 13 High P4 (13H), Day 14 Normal P4 (14N), Day 14 High P4 (14H).

**Figure 6:**
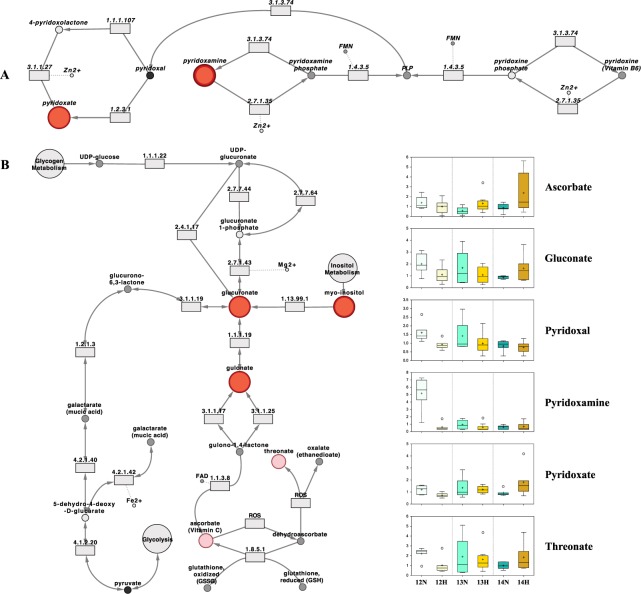
The (**A**) pyridoxal (vitamin B6), and (**B**) ascorbate (vitamin C) metabolic pathways in addition to the scaled intensities of relevant biochemicals. Biochemical node colour corresponds to day by progesterone (P4) interaction – dark red indicates a significant (p ≤ 0.05) interaction (node border thickness is inversely proportional to the magnitude of the p-value). Within these statistically significant nodes, node diameter is correlated to metabolic hierarchy (cofactor and intermediate metabolite nodes are smallest, followed by by-products, and central metabolites). Light red depicts an increasing trend (0.05 < p < 0.10), black depicts an identified amino acid which did not exhibit a day by progesterone interaction, and grey represents a biochemical present in the metabolic library but not detected in this study. Box plots: The y-axes are the relative metabolite concentrations, with the central horizontal line representing the median value with outer boundaries depicting the upper and lower quartile limits. Error bars depict the minimum and maximum distributions, with + representing the mean value and ○ the extreme data point. Abbreviations: Day 12 Normal P4 (12N), Day 12 High P4 (12H), Day 13 Normal P4 (13N), Day 13 High P4 (13H), Day 14 Normal P4 (14N), Day 14 High P4 (14H).

**Figure 7:**
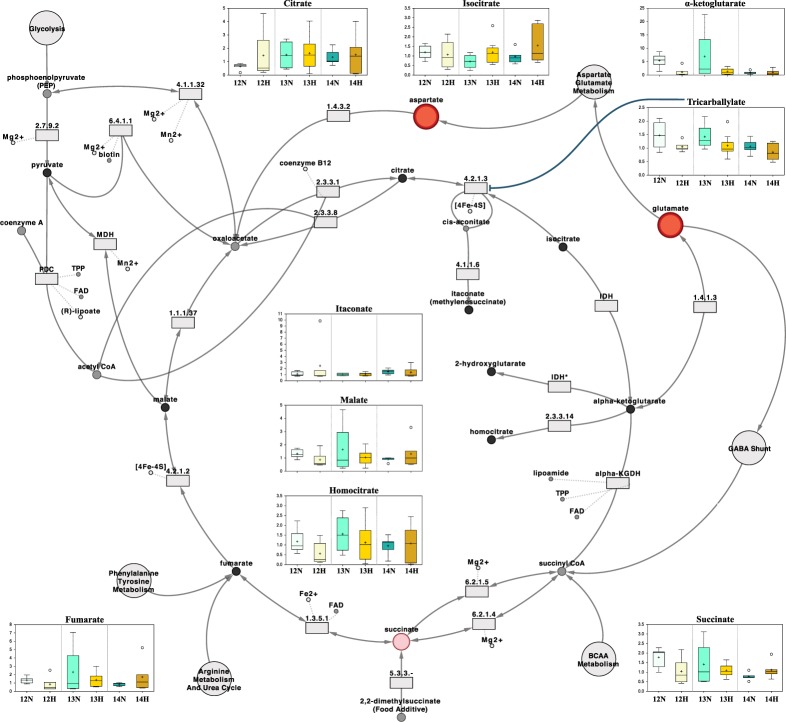
Tricarboxylic acid (Krebs) cycle metabolism, surrounded by the scaled intensities of relevant biochemicals. Biochemical node colour corresponds to day by progesterone (P4) interaction – dark red indicates a significant (p ≤ 0.05) interaction (node border thickness is inversely proportional to the magnitude of the p-value). Within these statistically significant nodes, node diameter is correlated to metabolic hierarchy (cofactor and intermediate metabolite nodes are smallest, followed by by-products, and central metabolites). Light red depicts an increasing trend (0.05 < p < 0.10), black depicts an identified amino acid which did not exhibit a day by progesterone interaction, and grey represents a biochemical present in the metabolic library but not detected in this study. Box plots: the central horizontal line represents the median value with outer boundaries depicting the upper and lower quartile limits. Error bars depict the minimum and maximum distributions, with + representing the mean value and ○ the extreme data point. Abbreviations: Day 12 Normal P4 (12N), Day 12 High P4 (12H), Day 13 Normal P4 (13N), Day 13 High P4 (13H), Day 14 Normal P4 (14N), Day 14 High P4 (14H).

**Figure 8:**
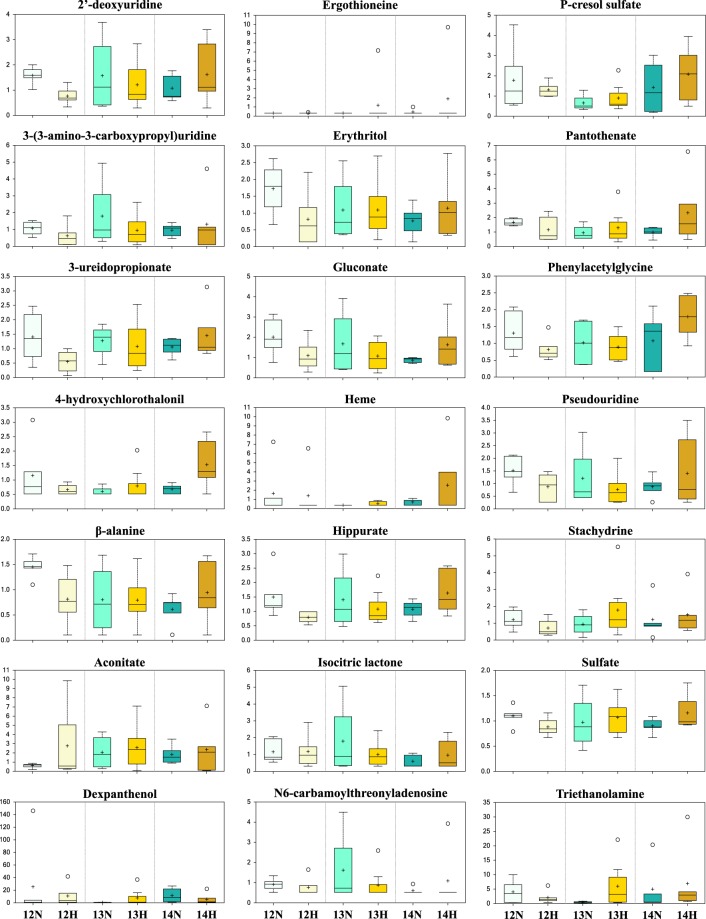
Scaled intensity boxplots of additional metabolites identified in this study. The y-axes are the relative metabolite concentrations, with the central horizontal line representing the median value with outer boundaries depicting upper and lower quartile limits. Error bars depict the minimum and maximum distributions, with + representing the mean value and ○ the extreme data point.

The implications of these findings are discussed below within the context of purine (Fig. 5), pyridoxal (vitamin B6), (Fig. 6A), ascorbate (vitamin C) metabolism (Fig. 6B), and the tricarboxylic acid cycle (Fig. 7), amongst other categories. Quantitative box-plots for remaining metabolites identified in this study are provided in Fig. 8.

## Discussion

This dynamic presence of energy metabolites, nucleotides, cofactors, vitamins, peptides, and xenobiotics in bovine uterine luminal fluid during the initiation of conceptus elongation offers new insights into maternal-embryo communication in ruminants. Key findings include: (*a*) elevated P4 is associated with metabolite suppression on Day 12, (*b*) biochemicals involved in nucleotide metabolism dominate the day by P4 interactions observed, and (*c*) metabolites constituting purine, pyrimidine, pyridoxal, ascorbate, and tricarboxylic acid cycle metabolism may play a central role in the conceptus elongation process. These are discussed below.

Nucleotides account for approximately 15–20% of non-aqueous cellular biomass^26^ and fulfil a plethora of central biological processes, including in DNA replication, RNA production, and protein synthesis, rendering them of likely importance to trophoblast proliferation and consequent conceptus elongation. Moreover, accelerated cellular proliferation requires rapid protein synthesis, for which cells require more ribosomes, in turn necessitating more ribosomal RNA (rRNA), and therefore, an increased accumulation rate of ribonucleoside triphosphates (rNTPs)^26^.

Whilst all cells have an inherent capacity for *de novo* nucleotide biosynthesis – a highly endergonic process – they can also uptake nucleotides^35,36^. Interestingly, all four ribonucleosides (adenosine, cytidine, guanosine, and uridine) were present in ULF, as were three of the corresponding ribonucleobases (adenine, guanine, and uridine). This finding, in conjunction with previous identifications of proteins relating to nucleotide transport and metabolism in ULF – *e.g*. polypyrimidine tract-binding protein 1 and bifunctional purine biosynthesis protein^37^ – suggests that uterine nucleotide release into ULF during the period of elongation initiation, is conducive to successful conceptus elongation, presumably for RNA production (Fig. 5); the RNA-exclusive nucleoside, uridine, displayed the second greatest day by P4 interaction, increasing in luminal abundance on Day 14 *vs*. 12 and 13 in the high P4 group, whilst thymine and thymidine, the respective DNA-exclusive nucleobase and nucleoside, were not identified.

Cofactors and vitamins was the only metabolite group displaying a significant flux when comparing all metabolites by treatment and day (Fig. 4). Regarding day by P4 interactions, the enrichment values for nicotinate and nicotinamide, vitamin B6, and ascorbate and aldarate metabolism were 2.5, 2.5, and 1.2, respectively. Nicotinamide is a key intermediate in NAD synthesis, which is crucial for energy metabolism (discussed below). Concentrations of both nicotinamide and nicotinamide riboside were lower in the ULF from high *vs*. normal P4 heifers on Day 12. Their concentrations, moreover, decreased in the normal P4 group from Day 12 to 14, suggesting decreased uterine nicotinamide salvage and metabolism in the high P4 heifers, likely as a result of altered epithelial energy metabolism. This may be important as a recent study conducted in humans and mice found the nicotinamide pathway plays an important role in miscarriage prevention^38^.

Retinol (vitamin A) presence was also interesting as it is involved in uterine epithelial cell differentiation regulation and matrix remodelling^39^. Retinol exhibited a P4 main effect by trending higher in the ULF of high P4 heifers on Days 12 and 14 relative to control. This finding is broadly in line with data from the porcine, wherein P4 increases uterine retinol binding protein (RBP) abundance^40^. Whilst RBP4 has been identified in bovine ULF^37^, further research is required to elucidate the roles of retinol and RBPs in ruminant maternal-embryo communication; not least as retinol inhibits activator protein 1 binding to the cyclic AMP response element of the COX-2 promoter, in turn, inhibiting COX-2 transcription, and reducing prostaglandin synthesis^41^. Moreover, it has been proposed that retinol contributes to uterine quiescence in the human myometrium during pregnancy^42^.

Similarly to nicotinamide and nicotinamide riboside, the concentration of all detected vitamin B6 metabolites (pyridoxamine, pyridoxal, and pyridoxate) was lower in high *vs*. normal P4 heifers on Day 12, and generally decreased over time in the normal P4 cohorts (Fig. 6). Interestingly, bacteria of the mammalian intestinal microbiome have the capacity to synthesise vitamins B3, B5, B6, and B12^43^. Given the day by P4 interaction displayed by pyridoxamine and pyridoxate, phenol sulfate^20^, and trend towards a day by P4 interaction by hippurate – all of which are microbiome-associated and unrelated metabolites – it is tempting to suggest that P4 may alter the bacterial profile of the bovine uterus^44^ and, in turn, ULF composition.

Further supporting this notion that ULF composition may be influenced by symbiotes is the identification of microbiome-associated^45,46^ xenobiotics ergothioneine and mannonate in ULF. Moreover, the presence of ascorbate (vitamin C), in addition to uric acid – both potent antioxidants^47^ – hints at the capacity of ULF to regulate redox homeostasis. These findings, taken together, highlight the potential role, and need for research into the impact, of factors beyond endometrial secretions on maternal-embryo communication.

Even though just two peptide metabolites were identified, the most pronounced day by P4 interaction observed in this study was that of γ-glutamylglutamine – a dipeptide of amino acids glutamate and glutamine, and proteolytic breakdown product^48^. The increase in γ-glutamylglutamine levels on Days 13 and 14 *vs*. 12 in high P4 heifers may be indicative of elevated uterine luminal protease^37^ activity. However, whether γ-glutamylglutamine, and phenylacetylglycine for that matter, are short-lived intermediates *en route* to further proteolysis, or have physiological cell-signalling effects, is unknown. It is worth noting the murine hatching embryo secretes a trypsin-like protease *in vitro*^49^, and, in the equine, peptidase D and the proteasome 26S subunit were in greater abundance in the ULF of pregnant *vs*. cyclic mares on Day 13^50^. Thus, uterine luminal protein degradation may be a free amino acid generation mechanism.

The TCA cycle is generally considered the central metabolic hub of the cell as it is the final common pathway for fuel molecule oxidation, including carbohydrates, fatty acids, and amino acids^51^. Forde *et al*.^37^ previously identified 5 of the 8 enzymes involved in the TCA cycle in bovine ULF: aconitase, isocitrate dehydrogenase, fumarate hydratase (fumarase), malate dehydrogenase, and citrate synthase. Here we identified 6 of the 10 key corresponding metabolites: citrate, aconitate, isocitrate, 2-oxoglutarate, succinate, and malate; raising the question of whether parts of the TCA cycle (Fig. 7) are active in ULF.

Of particular intrigue is α-ketoglutarate (*a.k.a*. 2-oxoglutarate), which exhibited a P4 main effect owing to a decrease in high *vs*. normal P4 heifers on Day 12 in addition to a decline in normal P4 heifers on Day 14 *vs*. 12 – *i.e*. 2-oxoglutarate levels *inversely* correlated with an advanced elongation environment. Whilst this intuitively implies that extracellular 2-oxoglutarate is not important for the elongating conceptus, it is telling that ULF is enzymatically equipped to produce 2-oxoglutarate from any one of malate, citrate, or isocitrate^37^. Aside from its role as a TCA cycle intermediate, 2-oxoglutarate is a common source of amino acids glutamate and glutamine^52^; though such a breakdown in ULF is unlikely as the catalyst, glutamate dehydrogenase, has not been identified in ULF. Nonetheless, 2-oxoglutarate can scavenge and sequester hydrogen peroxide (H_2_O_2_) *in vitro* prior to succinate conversion^53^. As H_2_O_2_ is a main source of reactive oxygen species, known to increase embryonic DNA damage^54^, further investigation into this phenomenon within an *in vivo* reproductive context is warranted.

Succinate followed an identical profile to 2-oxoglutarate and trended towards a day by P4 interaction. It is important to highlight that succinate synthesis would be the terminal step in a ULF TCA cycle, as succinate dehydrogenase [*a.k.a*. succinate-coenzyme Q reductase (SQR) or respiratory complex II], which catalyses the reversible oxidation of succinate to fumarate, is solely localised at inner mitochondrial membranes^55^. Regardless, succinate plays numerous signalling roles, such as stabilizing the hypoxia-inducible factor-1 alpha (HIF-1α) transcription factor^56^, whose knockout in the mouse is embryo-lethal owing to vascular defects in the embryonic and extraembryonic vasculature^57^. Moreover, G-protein coupled receptor 91 (GPCR91), previously considered an orphan receptor^58^, has been recently shown to sense extracellular succinate^59^, and *in utero* upregulation of GPCR91 in the rat foetus predisposes the offspring to hypertension^60^.

Given that all primary constituents of the first TCA cycle reactions have been identified in ULF, it is plausible to assume a role for energy metabolites in conceptus elongation, perhaps beyond energy production. Moreover, the presence of tricarballylate – an aconitase (the enzyme catalysing the formation of isocitrate from citrate) inhibitor^61^ of aerobic bacterial origin^62^ – further exemplifies the need to investigate the impact of the microbiome in uterine metabolism and maternal-embryo communication.

Furthermore, whilst this work on maternal-embryo communication using cyclic heifers inherently focuses on a unidirectional monologue (maternally secreted metabolites), future work includes similarly analysing ULF from pregnant heifers – in addition to corresponding conceptus-conditioned culture medium – to tease out the reciprocity of bovine pregnancy establishment. Additional future work includes further investigating P4-induced changes in the ruminant ULF proteome. For example, uterine serpins (or milk proteins) are secreted by the ovine^63–65^ and bovine^66^ uterus, in a hormonally responsive manner, presumably as a mechanism of immunomodulation, despite their classification as proteinase inhibitors^67^. Similarly, elucidating the precise role of conceptus-derived proteins, such as trophoblast Kunitz domain proteins – also categorised as proteinase inhibitors^68^ – is essential to furthering our understanding of maternal-embryo communication and pregnancy establishment in ruminants.

Lastly, it is important to note that elevated P4 on Day 5, but not thereafter, in the high P4 group is consistent with previous observations of accelerated conceptus elongation on Days 12–14^14^. More specifically, elevated P4 resulting from PRID introduction on Day 3 temporally advances the endometrial transcriptome by ~48–72 h during the conceptus elongation window^69^ despite systemic P4 concentrations returning to normal^12^.

In conclusion, this manuscript presents and describes the dynamic presence of energy substrates, nucleotides, cofactors, vitamins, and xenobiotics in the bovine uterine luminal fluid during the conceptus elongation-initiation window. The data show that elevated P4 suppresses ULF metabolite concentrations on Day 12 and may alter the uterine microbiome. Moreover, ULF has the capacity to act as a redox buffer, and biochemicals in ULF involved in purine, pyrimidine, pyridoxal, ascorbate, and tricarboxylic acid cycle metabolism likely facilitate conceptus elongation initiation.

## Methods

Sample collection details are described in detail in Simintiras *et al*.^20^, a summary of which is provided below.

### Animals and experimental design

Animal work was approved by the University College Dublin Animal Research Ethics Committee and licensed by the Irish Health Products Regulatory Authority, with experimentation performed in line with the European Community Directive 2010/63/EU. The estrous cycles of Charolais and Limousin crossbred heifers with a mean age (±SD) of 24.9 ± 5.6 months and weight (±SD) of 601.6 ± 47.7 kg were synchronized (n = 35) with a gonadotropin releasing hormone (GnRH) analogue (Ovarelin, Ceva Santé Animale) injection, followed by P4-releasing intravaginal device (PRID; Ceva Santé Animale) insertion. After 7 days, a prostaglandin F2α (PGF2α) analogue (Enzaprost; Ceva Santé Animale) was administered, and PRIDs were removed the next day. On Day 3 post-estrus, 20 randomly allocated heifers received another PRID until slaughter on Days 12–14 (thus, the high P4 group). The remaining 15 animals comprised the normal P4 group. Specifically, the experimental groups were: (***i***) Day 12 normal P4 (n = 6), (***ii***) Day 12 high P4 (n = 6), (***iii***) Day 13 normal P4 (n = 4), (***vi***) Day 13 high P4 (n = 8), (***v***) Day 14 normal P4 (n = 5), and (***vi***) Day 14 high P4 (n = 6).

### Uterine luminal fluid recovery

The uterine horn ipsilateral to the corpus luteum was excised and flushed with 10 ml phosphate buffered saline (PBS; Sigma Aldrich) within 60 mins of slaughter, prior to centrifugation for 15 min at 1000 × *g*. The supernatant was aliquoted, snap-frozen in liquid nitrogen, and stored at −80 °C until analysis.

### Progesterone analysis

Blood was collected from all heifers by coccygeal venepuncture on Days 3 and 5 in addition to the morning of slaughter, stored at 4 °C for 24 h, and centrifuged at 1500 × g for 20 min at 4 °C. The supernatant was stored at −20 °C until analysis. P4 was quantified by solid-phase radioimmunoassay (PROG-RIA-CT, DIAsource) in line with manufacturer guidelines, and as described in Simintiras *et al*.^20^.

### Metabolomics and data extraction

Metabolon Inc. performed the sample preparation and analysis by ultrahigh performance liquid chromatography-tandem mass spectroscopy (UPLC-MS/MS). Briefly, all samples were analysed by four reverse-phase (RP/UPLC)-MS/MS methods involving positive and negative ion mode electrospray ionization, in addition to hydrophilic interaction chromatography UPLC-MS/MS. Metabolites were identified by retention time and a *m*/*z* within ± 10 ppm, and quantified against known internal and recovery standards, which were run in parallel at random intervals. Data were corrected for inter-day instrument tuning variations – median peak areas for each metabolite were registered as 1.00 prior to the proportional normalisation of each data point. Data were then logarithmically transformed and quantified by relative abundance using MetaboLync pathway analysis (MPA) software (portal.metabolon.com) and visualized using Java Cytoscape 3.6.1. Relative mean metabolite concentrations were calculated by averaging the median scaled imputed data of all metabolites for each aforementioned experimental group.

### Data analysis and statistics

Semi-quantitative metabolomic data (either expressed as main effects, interactions, fold-changes, or relative concentrations), were statistically analysed by two-way ANOVA with a p ≤ 0.05 or 0.05 < p < 0.10 cut off. Where stated, a Holm-Sidak non-parametric *post hoc* test was also used. All samples described under ‘animals and experimental design’ (total n = 35) were included in the analyses.

### Pathway enrichment

The measure of intra-pathway metabolite flux relative to inter-pathway metabolite flux [pathway enrichment (E)] was calculated using the following formula: (*k*/*m*)/(*n*/*N*) where *k* = number of significant metabolites per pathway, *m* = total detected metabolites per pathway, *n* = number of significant metabolites in the study, and *N* = total identified metabolites in the study. A value > 1 indicates pathway enrichment, and a value < 1 denotes pathway under-enrichment, whereas a value of 0 indicates the pathway was unenriched.
